# Investigation of immunomodulatory and cytotoxic effects of shed snake skin (*Elaphe sauromates*) extract

**DOI:** 10.3389/fphar.2024.1270970

**Published:** 2024-07-12

**Authors:** Cagri Caglar Sinmez, Emre Tüfekçi, Büşra Şeniz Demir, Ahmet Eken, Vehbi Guneş, Seda Ekici, Esra Bozkaya, Ali İlteriş Aykun

**Affiliations:** ^1^ Department of History of Veterinary Medicine and Deontology, Faculty of Veterinary Medicine, Ziya Eren Drug Research and Application Center (ERFARMA), Erciyes University, Kayseri, Türkiye; ^2^ Department of Internal Medicine, Faculty of Veterinary Medicine, Erciyes University, Kayseri, Türkiye; ^3^ Department of Medical Biology, School of Medicine, Betül-Ziya Eren Genome and Stem Cell Center (GENKÖK), Erciyes University, Kayseri, Türkiye; ^4^ Department of Internal Medicine, Faculty of Veterinary Medicine, Experimental Research and Application Center (DEKAM), Kayseri, Türkiye; ^5^ Veterinary Control Central Research Institute, Ankara, Türkiye; ^6^ Scientific and Technological Research Application and Research Center, Kırıkkale University, Kırıkkale, Türkiye; ^7^ Department of History of Veterinary Medicine and Deontology, Faculty of Veterinary Medicine, Erciyes University, Kayseri, Türkiye

**Keywords:** Shed snake skin, immunomodulation, immunity, pro-inflammatory cytokines, T lymphocytes

## Abstract

**Introduction:**

Shed snake skin (SSS) is commonly used empirically in ethnomedicine to treat psoriasis, acne, warts, eczema, scabies, open wounds, hemorrhoids, and glaucoma. Although a few studies exist, SSS extracts’ *in vitro* immunological effects have yet to be well described. Therefore, we aimed to investigate the immunomodulatory effects of SSS extract on murine lymphocytes and T cells.

**Methods:**

Hexane, methanol, and chloroform extractions were conducted in collected SSS samples. Protein concentrations in the SSS extract were measured. The cytotoxic and anticancer activities were measured using L929 Fibroblast and SK MEL 30 Cell Lines via MTT assay as described in TS EN ISO 10993-5. Immunomodulatory activities of SSS extract on total lymphocytes or enriched CD4^+^ T cell cultures, their cell-specific pro-inflammatory cytokines (*IL-6*, *IL-1β*. *IL-12p40*, *IL-23p19*, *TNF-*α, *IL-17A*, *IFN-γ*, *IL-10*, *TGFβ1*) levels were measured via FACS ARIA III analysis and related gene expression with Real-Time Quantitative Polymerase Chain Reaction (Rt-qPCR).

**Results:**

Hexane, methanol, and chloroform-extracted SSS were tested on SK-MEL-30 cells via MTT and revealed a superior anti-proliferative effect for hexane extract of SSS at low concentrations. SSS treatment of murine lymphocytes augmented Tnf-α and IFN-γ levels produced by CD3^+^ T cells when lymphocytes were activated with anti-CD3/CD28 or LPS stimulation. This effect required the presence of non-T cells, possibly antigen-presenting cells, and was not observed on purified CD4^+^ T cells. Additionally, SSS significantly blocked suppressive cytokine *Tgfb* gene expression (but not *Il10*) without altering *in vitro* Treg generation/or expansion.

**Discussion:**

This is the first *in vitro* study investigating SSS’s anti-tumor and immunomodulatory effects. Our data provide evidence for SSS’s anti-proliferative activity on SK-MEL-30 cells and its pro-inflammatory role on murine lymphocytes, which warrants further investigation of the potential use of SSS extract with *in vivo* disease models.

## 1 Introduction

Snakes undergo a phenomenon commonly referred to as “shedding” or “skin changing”, which occurs approximately 3-4 times annually or at least once a year during specific phases of their life cycle (Greene, 1997). Shed snake skin (SSS) constitutes a multi-layered membrane composed of bioactive keratin and lipids, serving as a natural source of mechanical support. The perpetually regenerating epidermis represents a significant and invaluable component of snake skin. Notably, during the shedding process, the older epidermal layer of snakeskin typically undergoes a complete molt facilitated by a complex mechanism. The snake skin structure comprises four distinct layers: the outermost layer, referred to as “Oberhautchen,” followed by the β layer, the “Mesos” layer enriched in lipids, and the thinner α layer, predominantly comprised of protein (Ripamonti et al., 2009).

SSS has a long history of empirical use in ethnomedicine for treating various conditions, including psoriasis, acne, warts, eczema, scabies, open wounds, hemorrhoids, and glaucoma (Greene, 1997; Orozobaev, 2012; Sakallı and Çömelekoğlu, 2019). For many years, the uses of SSS in traditional Chinese medicine include a variety of medical conditions such as furuncles, carbuncles, breast abscesses, and even inguinal hernias (Lev, 2003; Sinmez, 2011; Mukherjee et al., 2012; Vakilian et al., 2019). It is also known that traditional healers in Southeast Asia and India have used SSS in the regulation of reproductive functions for many years (Costa-Neto, 2005). The application of SSS in treating these ailments has traditionally been believed to yield successful therapeutic outcomes. SSS has been considered a valuable resource in traditional medicine due to its potential medicinal properties. The belief in its efficacy has led to its application in treating various dermatological conditions and even eye-related ailments such as glaucoma (Greene, 1997; Lev, 2003; Sinmez, 2011; Orozobaev, 2012; Sakallı and Çömelekoğlu, 2019). Additionally, according to findings from ethnoveterinary medicine studies, the oral administration of SSS to cure papillomatosis in cows is an effective treatment (Sinmez, 2011).

The papillomatosis cases in cattle are known to be associated with skin lesions, which are thought to be caused by the bovine papillomavirus (Sakallı and Çömelekoğlu, 2019). Therefore, the antiviral-immunity activity of SSS may be related to its therapeutic efficacy, as demonstrated in ethnoveterinary medicine investigations. Anti-viral immunity relies on the Type I immune module, which involves M1 macrophages, dendritic cells, NK cells (Björkström et al., 2022), CD8^+^ T cells (Cox et al., 2013), and CD4^+^ Th1 (Kervevan and Chakrabarti, 2021) cells along with complement-inducing IgG antibody responses. IL-12 and IL-18 are the inducers, and IFN-γ, Tnf-α, and IL-2 are the effector cytokines of this immune module (Cox et al., 2013; Kervevan and Chakrabarti, 2021; Björkström et al., 2022).

Eczema and psoriasis immunopathogenesis involve heightened Type II and Type I immune modules, which include Th2 and Th17 cells, respectively (Vičić et al., 2021; Makowska et al., 2023). Type II immunity includes M2 macrophages, ILC2s as inducers, Th2, Tfh, and eosinophils\ basophils, and mast cells as effectors that work through IL-4, IL-5, IL-13 signature cytokines. Type III immune module involves ILC3s, Th17 cells, and neutrophils and works through IL-17A/F, IL-22, GM-CSF, and antimicrobial peptides. All three immune modules contribute to protective immunity against intracellular and extracellular pathogens, helminths and parasites, fungal pathogens, and tumors (Murphy and Weaver, 2017).

Despite its use in folk medicine, there needs to be more literature regarding the immunomodulatory features of SSS and its potential impact on the immune modules. However, more experimental studies are required for SSS in the literature. Mukherjee et al. (2012) conducted a study to determine that the aqueous skin extract of the Indian cobra (Naja naja), which is frequently used in female reproductive disorders in India, was influential on the estrous cycle in female Swiss albino mice, especially in the diestrus cycle, causing the estrous cycle to end. They also reported that SSS affected the reproductive cycle of mice through a hormone-cytokine exchange (Mukherjee et al., 2012). Mukherjee et al. (2016) isolated NNSS2 from the SSS of the Indian cobra (Naja naja). They explained that this small peptide called NNSS2 is effective in the estrous cycle (Mukherjee et al., 2016). Ghosh et al. (2022) conducted a study in which an extract prepared from the shed skin of the Naja Naja Indian cobra was intraperitoneally injected into male mice at a dosage of 10 mg/kg for 7 days using physiological saline. The study revealed significant alterations in the overall histological structure of the seminiferous tubules. These alterations included irregularities in germinal epithelial cells, the accumulation of exfoliated cells in the tubule’s lumen, the presence of tailless sperm in the epididymis, as well as irregularities in basal cells within the vas deferens. Furthermore, notable changes were observed in sperm morphology (Ghosh et al., 2022).

On the other hand, Sakallı and Çömelekoğlu (2019) found that SSS of the black snake (*Dolichopis jugular*) *and pithead snake* (*Malpolon insignis*) species are especially rich in trace elements (Zn, Cu, Ti, Mn, Fe, Sr, Ba, Ni). Vakilian et al. (2019) investigated the wound-healing effect of the shed skin of two different Omani snakes in rats (Vakilian et al., 2019). Researchers have shown that SSS can heal acute wounds directly or indirectly and help release inflammatory cytokines, resulting in epithelialization, neovascularization, and fibroblast expansion of granulation tissue formation. Findings obtained from studies conducted to date show that SSS is not only a biological waste but also can be an essential medical resource that contains chemical compounds with many valuable biological activities.

In new drug development studies in modern medicine, ethnobiological data have been used starting from the traditional methods (Costa-Neto, 2005). Thus, it is important to know the potential of SSS extract whose impact on immune cells have been largely unexplored. Therefore, this current study aimed to investigate the cytotoxic and possible immune modulatory activities of the SSS extract on murine lymphocytes obtained from C57BL6 mice upon activation of lymphocytes or sorted CD4^+^ T cells in culture with anti-CD3/CD28, phytohaemagglutinin, or lipopolysaccharide (LPS). The impact of SSS extract on cytokine production (IL-6, IL-1β, IL12p40, IL23p19, TNF-α, IL-17A, IFN-γ, IL-10, Tgfβ1) involved in innate and adaptive immunity have been assessed at the protein and/or mRNA level.

## 2 Materials and methods

### 2.1 Extraction of the collected shed snake skin

Ventral portions of shed skins were obtained from *Elaphe sauromates* (ES), which the Anatolian Wonderland Zoo, Kayseri, supplied. Dr. Coşkun TEZ identified the shed skins at Erciyes University’s Department of Zoology. The Erciyes University Local Ethics Committee for Animal Experiments (03 May 2023; Number: 2023-108) approved this study.

The obtained SSS were extracted using hexane, methanol, and chloroform solvents. For the hexane extract, 10 g of SSS were weighed, and 140 mL of hexane (Sigma-Aldrich) was added. The mixture was then placed in Soxhlet (BUCHI B-811, Switzerland), and the extraction process was conducted. The hexane extract was concentrated in a rotary evaporator (BUCHI R-210, Switzerland). The same procedures were performed using methanol and chloroform solvents, and three different extracts were obtained. The endotoxin levels in the extracts have been measured by Limulus Ameobocyte Lysate (LAL) assay, and found to be negative (detection limit 0.25 EU/mL).

### 2.2 Protein concentration analysis from shed snake skin extract

Five mg SSS extract was weighed. The extract was dissolved in a mixture containing 7-8 µL DMSO in 1 mL distilled water (containing 1% DMSO in the final volume) and vortexed. Total protein concentration was analyzed by the BCA method.

### 2.3 Investigation of cytotoxic effect and anticancer activity of shed snake skin extracts

L929 fibroblast and SK-MEL-30 cell lines (Human melanoma cell) were used to determine the cytotoxic effect and anticancer activity of hexane, methanol, and chloroform extracts of SSS. MTT assay, according to TS EN ISO 10993-5 standard, was used to determine cell viability. The MTT assay, also known as 3, [4,5-dimethylthiazol-2-yl]-2,5-diphenyltetrazolium bromide salt (Merck, CAS Number: 298-93-1), is a sensitive method used to measure mitochondrial dehydrogenase activity and is a colorimetric determinant of cell proliferation and metabolism.

SK-MEL-30 and L929 fibroblast cells were cultured in 96-well plates with 1.0 × 10^4^ cells in each well. Cells were incubated for 24 h. Hexane (Merck, CAS Number: 110-54-3), methanol (Merck, CAS Number: 67-56-1), and chloroform (Merck, CAS Number: 67-66-3) extracts were prepared at 2 mg/mL after drying. The extracts were applied to SK-MEL-30 cells at five different concentrations (2 mg/mL- 1 mg/mL- 0.5 mg/mL- 0.25 mg/mL- 0.12 mg/mL), and six different concentrations (2 mg/mL- 1 mg/mL- 0.5 mg/mL- 0.25 mg/mL- 0.12 mg/mL- 0.062 mg/mL). Then, they were incubated at 37°C 5% CO_2_ for 24 h, with the only medium as a blank. At the end of 24 h, the medium in the wells was discarded, and 50 μL of MTT (1 mg/mL) solution was added to each well. After incubation at 37°C for 2 hours, 100 μL isopropanol (Merck, CAS Number: 67-63-0) was added to the wells, and the absorbance values of the 96-well plate were read at 570 nm on a microplate reader to determine cell viability. Based on the control (blank) groups, % viability was calculated according to the following equation:

$$\%\text{Cell viability}=\frac{100\times {\text{OD}}_{570e}}{{\text{OD}}_{570b}}$$

*OD_570e:_ the value of the optical density of the sample*.

*OD*_*570b*:_ *optical density of control (blank) group*

### 2.4 Determination of apoptotic index of shed snake skin extracts applied to L929 fibroblast and SK MEL 30 cell lines

For double staining, L929 fibroblast and SK MEL 30 cells were seeded in 48 well plates with 20 × 10^3^ cells in each well and incubated for 24 h. At the end of 24 h, hexane, chloroform, and methanol extracts (2 mg/mL- 1 mg/mL- 0.5 mg/mL- 0.25 mg/mL- 0.12 mg/mL) of SSS were applied at indicated concentrations and left to incubate for 24 h. At the end of 24 h, fluorescent staining with Hoechst 33,342 and ribonuclease A was performed to determine the apoptotic index. Hoechst staining (33,342) stains cell nuclei blue. Although it stains all cells, apoptotic cells appear brighter, and the nucleus appears disorganized and fragmented, with a loss of homogeneity. This distinguishes actual apoptotic cells from normal cells (Melekoğlu et al., 2020).

### 2.5 Lymphocyte cell isolation and culture

Lymph nodes (inguinal, brachial, axillary) were harvested from 8-weeks old C57BL/6 and macerated with a syringe plunger over a 70 µm strainer (Cat no: 542,070) to obtain single cells in Phosphate Buffered Saline (PBS) solution, after which spun for 5 minutes at 400 g. Cells were obtained after disposing of the supernatant. After resuspension in complete RPMI (Cat no: 01-100-1A) (including %10 FBS (BI, cat no: 04-001-1A), L-Glutamine, Non-essential amino acid, Gentamicin, Penicillin/Streptomycin, all from Gibco) cells were used for experiments. CD4^+^ T cells were purified for some experiments using CD4 microbeads (MojoSort™ Mouse CD4 T Cell Isolation Kit, #480006, BioLegend) at ∼95% purity. Sorted CD4^+^ T cells were used for apoptosis, cytokine, and Treg differentiation experiments.

Cells were seeded into a 96-well round bottom plate as 1 × 10^6^ cells/well. Some wells were stimulated with Lipopolysaccharide (LPS) 1 μg/mL, others with CD3/CD28 1 μg/mL (of each). SSS extracts were added at three different concentrations (1 ng/mL, 0.1 μg/mL, and 1 μg/mL). The cell culture was incubated for 24 h at 37°C, 5% CO_2_.

### 2.6 Intracellular cytokine staining (ICC)

Following 24 h of stimulation with LPS (1 μg/mL) or anti-CD3 (1 μg/mL), the cells (either total lymphocytes or sorted CD4^+^ T cells) were stimulated with 50 ng/mL PMA (Sigma, cat no: P1585), 1 μg/mL Ionomycin (Sigma, Cat no: I3909), and 1 µL Golgi Stop (BD, Cat no: 554,715), and incubated for four extra hours at 37°C with %5 CO_2_. The cells were washed with PBS. Surface antibodies were added according to the manufacturer’s recommendation, and cells were incubated in the dark for 30 min at 4°C. After incubation, cells were washed twice with PBS with 2% FBS (Staining Buffer). BD Cytofix/Cytoperm™ Plus (554,715) was used for fixation and permeabilization per the manufacturer’s protocol. ICC antibodies were added to cells according to the manufacturer’s recommendation. Cells were washed with Staining Buffer twice. Samples were analyzed using BD FACs AriaIII.

The following antibodies were used: APC Anti-Mouse CD3 Antibody (Cat No: 100,236), PerCp/Cy5.5 Anti-mouse *IL-22* Antibody (Cat no: 516,411), FITC Anti-mouse GM-CSF Antibody (Cat no: 505,403), APC/Cyanine7 Anti-mouse IL-17 Antibody (Cat no: 506,919), Pacific Blue Anti-mouse IFN-γ Antibody (Cat no: 505,818), PE Anti-mouse IL-2 Antibody (Cat no:500,324), PE/Cyanine7 Anti-mouse Tnf-α Antibody (Cat no:3131715).

### 2.7 Lymphocyte apoptosis staining

Apoptosis staining was performed with FITC Annexin V Apoptosis Detection Kit (Cat:640922) according to the manufacturer’s recommendation. The lymphocytes or sorted CD4^+^ T cells were cultured in complete medium RPMI 1640 with 10% FBS, with or without stimulation with anti-CD3/28, (1 μg/mL each) after cells were exposed to the indicated concentrations of SSS for 24 h at 37°C with %5 CO_2_.

### 2.8 Real-time quantitative polymerase chain reaction (Rt-qPCR)

Cultured and overnight stimulated (with anti-CD3, 1 μg/mL; or phytohemagglutinin (PHA, Sigma, #11082132001) 5 μg/mL or LPS 1 μg/mL) lymphocytes obtained from C57BL/6 mouse lymph nodes or sorted CD4^+^ T cells were lysed with NucleoGene Tri Reagent (Cat no: NGE023) and RNA extraction was performed according to the instructions of the manufacturer. cDNA synthesis was performed using OneScript^®^ Plus cDNA synthesis kit (Cat no:#G236) from Abm. RT-qPCR was performed using SYBR Green (Cat no: #1725121). Gene expression was normalized over RPL19 using the delta (delta Ct) (2^−ΔΔCT^) method. The relevant genes and the primers used are given in Table 1.

**TABLE 1:** Nucleotide sequences of related genes used in real-time quantitative polymerase chain reaction.

Genes	Primers
***Rpl19***	FW 5′-CCA​ATG​CCA​ACT​CCC​GTC​A-3′
	RV 5′-TCT​TCT​TGG​ATT​CCC​GGT​ATC​T-3′
***ll6***	FW 5′-ACC​AGA​GGA​AAT​TTT​CAA​TAG​GC-3′
	RV 5′-TGA​TGC​ACT​TGC​AGA​AAA​CA-3′
***ll1b***	FW 5′-GCC​CAT​CCT​CTG​TGA​CTC​AT-3′
	RV 5′-AGG​CCA​CAG​GTA​TTT​TGT​CG-3′
***ll12p40***	FW 5′-TGG​TTT​GCC​ATC​GTT​TTG​CTG-3′
	RV 5′-TGG​TTT​GCC​ATC​GTT​TTG​CTG-3′
***Il23p19***	FW 5′-CCAGCGG GACATATGAATCT-3′
	RV 5′-AGG​CTC​CCC​TTT​GAA​GAT​GT-3′
***Tnfα***	FW 5′-TGC​CTA​TGT​CTC​AGC​CTC​TT-3′
	RV 5′-GAG​GCC​ATT​TGG​GAA​CTT​CT-3′
***Il17A***	FW 5′-CAT​GAG​TCC​AGG​GAG​AGC​TT-3′
	RV 5′-GCT​GAG​CTT​TGA​GGG​ATG​AT-3′
***Ifng***	FW 5′-TCA​AGT​GGC​ATA​GAT​GTG​GAA​GAA-3′
	RV 5′-TGGCT CTGCAGGATTTTCATG-3′
***ll10***	FW 5′-CCC​TTT​GCT​ATG​GTG​TCC​TT-3′
	RV 5′-TGGTTTCTCTTCCCA AGACC-3′
***Tgfβ1***	FW 5′-CTC​CCG​TGG​CTT​CTA​GTG​C-3′
	RV 5′-GCC​TTA​GTT​TGG​ACA​GGA​TCT G-3′

### 2.9 Regulatory T cell differentiation

CD4^+^ T cells were sorted from C57BL/6 mouse lymph nodes. The 96-well round bottom plates were precoated with anti-CD3 and CD28 for 2 hours at 37°C. Then, one hundred µL of PBS containing 2 μg/mL anti-CD3 and 1 μg/mL anti-CD28 was added to cover the bottom of the wells. The plates were washed twice with 100 µL PBS, and the sorted CD4^+^ T cells were seeded with 5 ng/mL recombinant mouse Tgfβ and 50 ng/mL recombinant IL-2 in complete RPMI 1640, containing FBS and antibiotics (Pen/Strep). SSS extract was added at indicated concentrations and refreshed on day 3. On the third day, 100 µL medium was replaced with fresh medium containing fresh cytokines (5 ng/mL Tgfβ and 50 ng/mL recombinant IL-2) and SSS. Five days later, the cells were first surface stained with CD4 (BioLegend, cat:100408) and then intracellularly with Foxp3 (BioLegend, cat:126408) using the transcription factor staining kit according to the manufacturer’s instructions (True-Nuclear™ Transcription Factor Buffer Set, BioLegend, cat:424401). The samples were run on FACS AriaIII and analyzed via FlowJoX.

### 2.10 Statistical analysis

Students’ t-tests or one-way ANOVA for normally distributed data were used where appropriate. For non-normally distributed data, non-parametric Kruskal Wallis and Dunn’s correction was used. GraphPad Prism nine was used for the analysis. A *p*-value of <0.05 was considered significant.

## 3 Results

### 3.1 Protein concentration analysis from shed snake skin extract

The total protein concentration was determined as 202.155 mcg/mL.

### 3.2 MTT cytotoxicity test results

**FIGURE 1:**
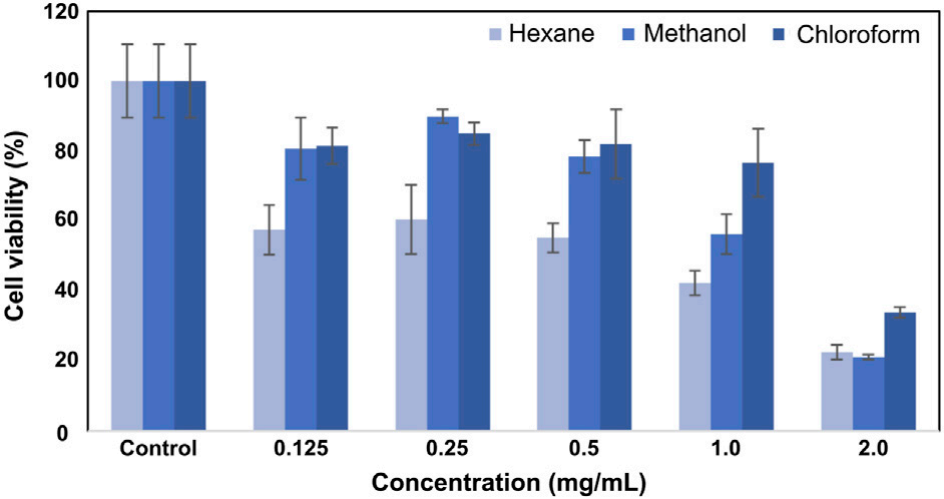
Viability plot of SK-MEL-30 cells treated with SSS extracts.

MTT test was used to determine the cytotoxicity of the extracts. The viability of SK MEL 30 cells treated with hexane, methanol, and chloroform extracts was calculated as 22.4 ± 2.1%- 21.1 ± 0.7%- 33.9% ± 1.5% at the highest concentration (2 mg/mL), respectively. Cell viability increased depending on the concentrations of methanol and chloroform extracts. In SK-MEL-30 cells treated with 0.12 mg/mL methanol and chloroform extracts, the viability was 80.7% ± 8.9% and 81.5% ± 5.2%, respectively. In cells treated with hexane extract, the viability was calculated as 57.4% ± 7.1%. According to MTT results, hexane extract was more effective than methanol and chloroform extracts on SK-MEL-30 cells and determined anticancer activity at low concentrations. IC50 values of hexane, methanol, and chloroform extracts were determined as 0.7 mg/mL, 1.2 mg/mL, and 1.6 mg/mL, respectively. The viability values of SK-MEL-30 cells treated with SSS extracts are shown in Figure 1.

**FIGURE 2:**
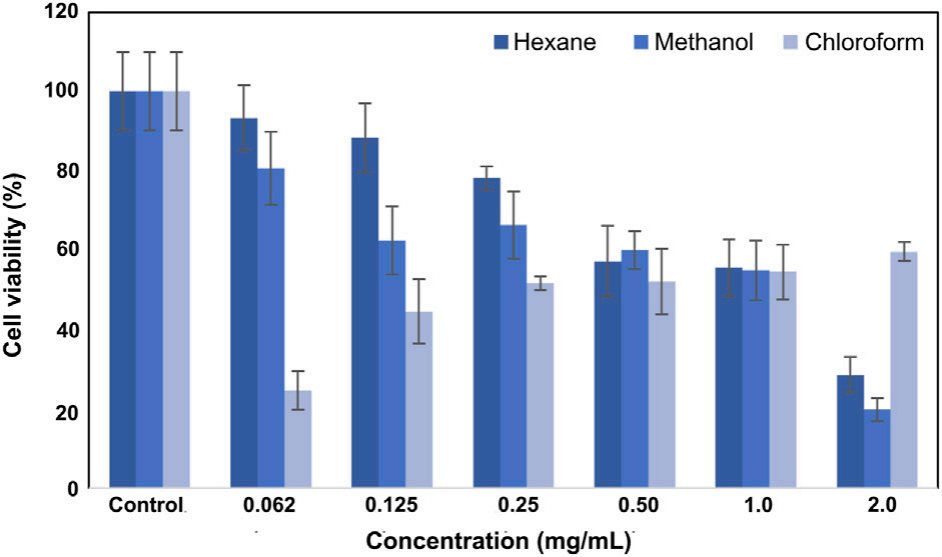
Viability plot of L929 fibroblast cells was treated with SSS extracts.

The viability values of L929 fibroblast cells after treatment with hexane, methanol, and chloroform extracts are given in Figure 2. Extracts at a concentration of 2 mg/mL showed high toxicity, and the viability at this concentration was calculated as 28.5% ± 7.2%, 19.8% ± 7.5%, and 24.6% ± 6.9%, respectively. As the applied concentration decreased, cell viability increased accordingly. Lower viability (44.6% ± 8.3%) was measured for cells treated with 1 mg/mL concentration. The highest viability was observed (88.3 ± 8.2) at a 0.12 mg/mL concentration. The viability values for L929 fibroblast cells after SSS extract treatment are shown in Figure 2.

### 3.3 Determination of apoptotic index

**FIGURE 3:**
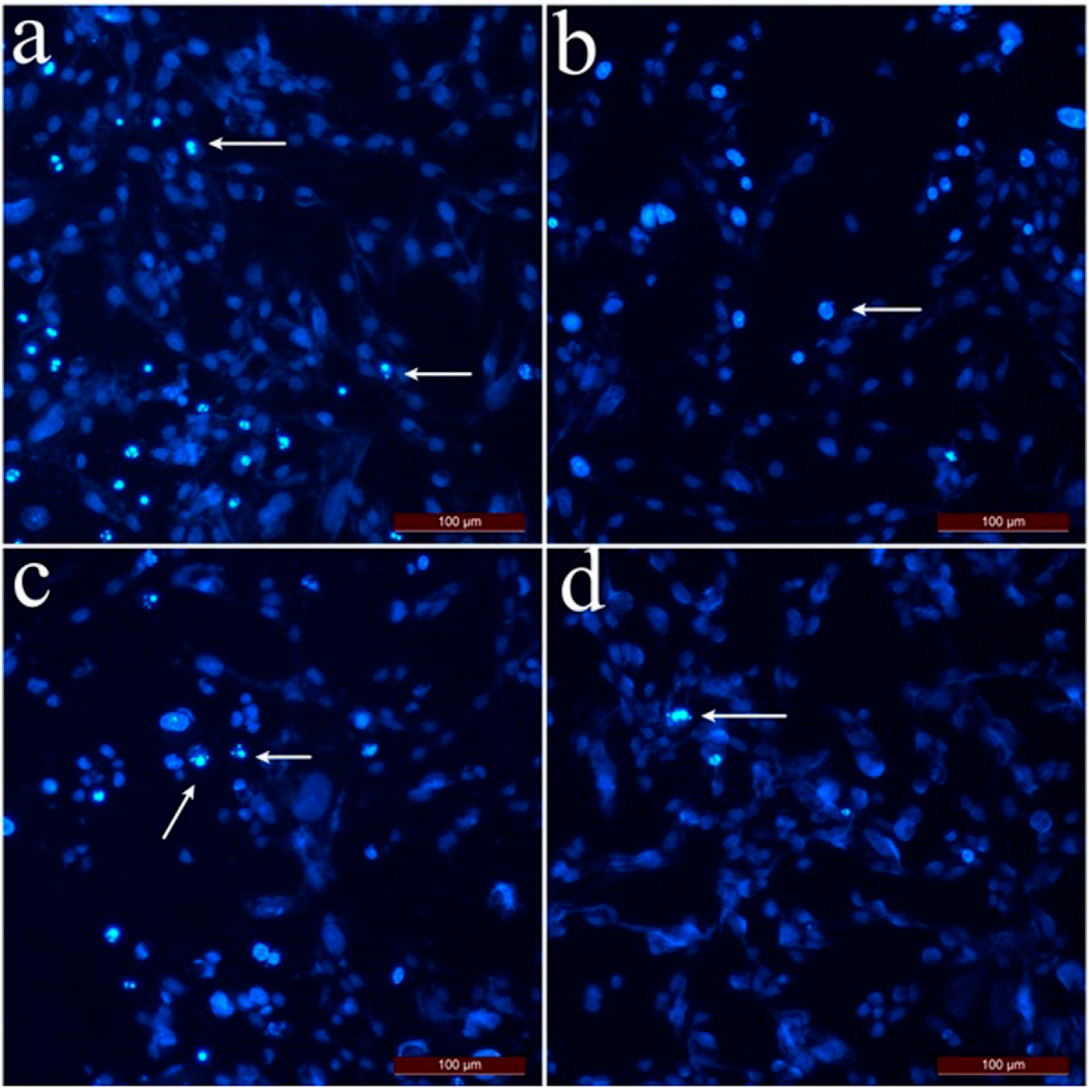
Dual staining images of SK-MEL-30 cells treated with SSS extracts. Arrows indicate apoptotic cells stained with Hoechst 33,342. **(A)** SK-MEL-30 cells were treated with hexane extract at 0.5 mg/mL concentration. **(B)** SK-MEL-30 cells treated with chloroform extract at 0.5 mg/mL concentration. **(C)** SK-MEL-30 cells were treated with methanol extract at 1 mg/mL concentration. **(D)** Control group cells.

According to the apoptotic index results determined by the dual staining method (Figure 3), the highest apoptotic index was calculated as 56.5% at 1 mg/mL concentration for SK-MEL-30 cells which were treated with hexane extract. For the cells treated with chloroform and methanol method extracts at a concentration of 1 mg/mL, the apoptotic index was calculated as 40.5% and 23.5%, respectively. The apoptotic index decreased depending on the concentration applied. The % apoptotic index values of SK MEL 30 cells treated with SSS extracts are shown in Figure 3.

**FIGURE 4:**
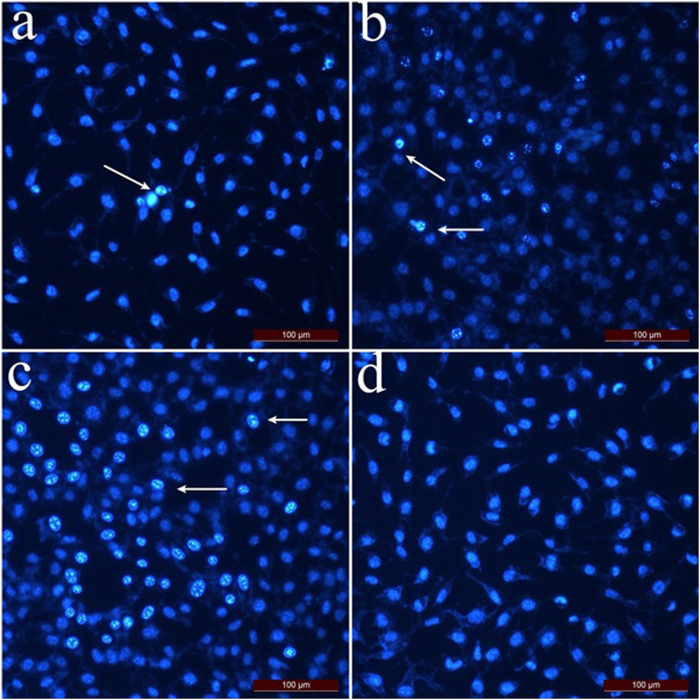
Dual staining images of L929 fibroblast cells treated with SSS extracts. Arrows indicate apoptotic cells stained with Hoechst 33,342. **(A)** L929 fibroblast cells were applied with hexane extract at 0.25 mg/mL concentration. **(B)** L929 fibroblast cells were applied with chloroform extract at 1 mg/mL concentration. **(C)** L929 fibroblast cells were applied with methanol extract at a 0.5 mg/mL concentration. **(D)** Control group cells.

The apoptotic index of L929 fibroblast cells (Figure 4) was calculated as 36.6% at 2 mg/mL concentration and 15.3% at 1 mg/mL concentration if the cells were treated with hexane extract. As the applied concentrations decreased, the apoptotic index for L929 fibroblast cells decreased compared to SK-MEL-30 cells. For L929 fibroblast cells treated with methanol and chloroform extract, the apoptotic index was 12.5% and 21.1% at 2 mg/mL concentrations, respectively. The % apoptotic index values of L929 fibroblast cells treated with SSS extracts are shown in Figure 4.

When the MTT cytotoxicity test and apoptotic index results were taken into consideration, the hexane extract of SSS showed an anti-proliferative effect even at low concentrations on SK-MEL-30 cells, and the apoptotic index was higher than chloroform and methanol extract. In addition, the high viability of L929 fibroblast cells at concentrations where hexane extract showed an antiproliferative effect in SK-MEL-30 cells indicates that hexane extract may have anticancer activity.

### 3.4 Apoptotic effects of SSS on C57/BL6 mouse lymphocytes

**FIGURE 5:**
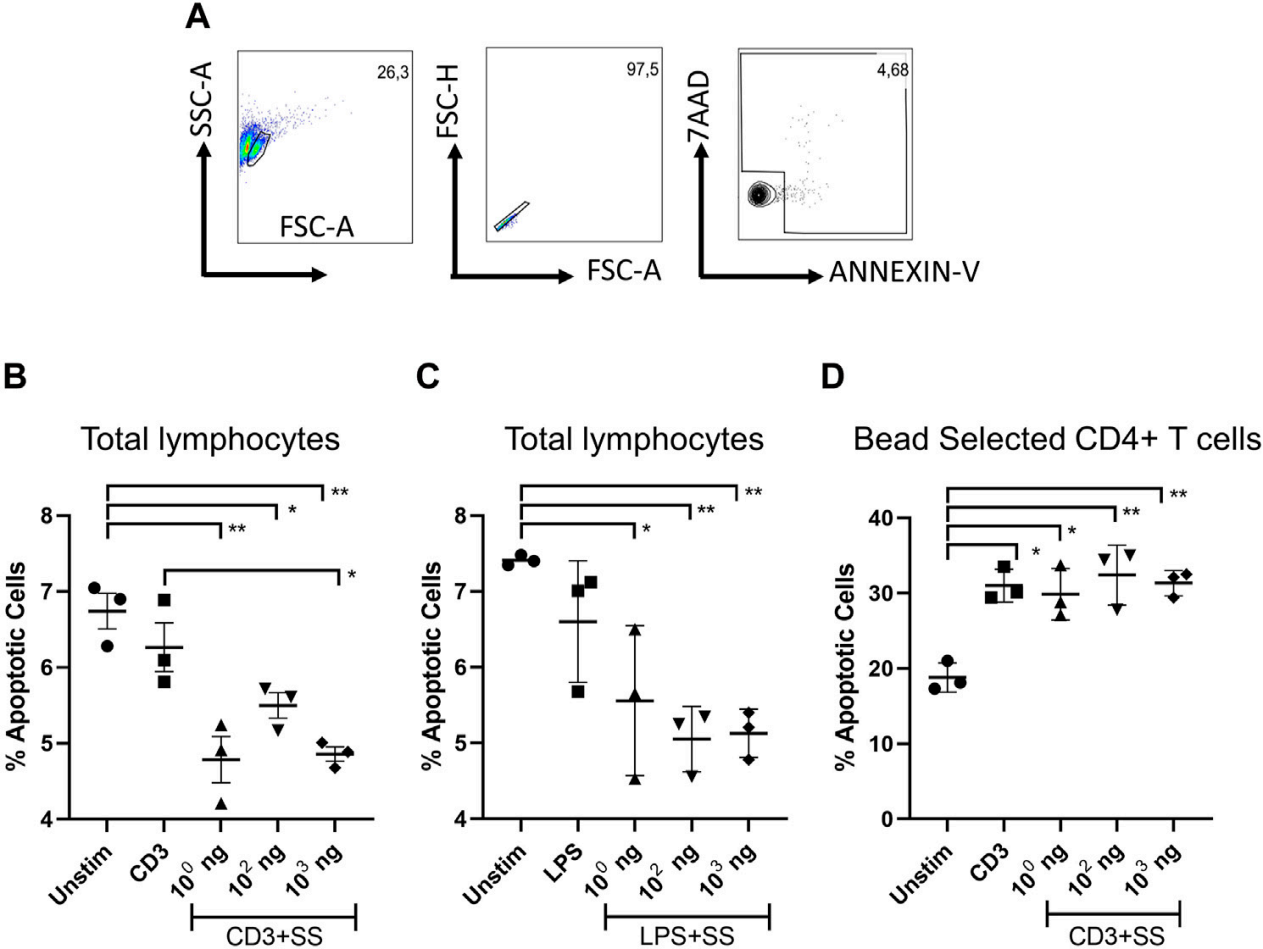
The impact of SSS on activation-induced lymphocyte cell death: **(A)** Flow cytometry gating strategy. The percentage of dying cells among LN lymphocytes (AnnexinV+, AnnexinV+7AAD+) **(B)** upon anti-CD3/CD28 activation (1 μg/mL each), or **(C)** LPS stimulation (1 μg/mL) for 24 h. **(D)** The percentage of dying cells among microbead selected CD4^+^ T cell (AnnexinV+, AnnexinV+7AAD+) **(B)** upon anti-CD3/CD28 activation (1 μg/mL each) The experiment was performed in technical triplicates with pooled lymph node lymphocytes from 1 mouse each time and repeated three times. (*) indicates *p* < 0.05, (**) indicates *p* < 0.01.

The apoptotic effect of SSS hexane extract on lymphocyte cultures obtained from C57BL6 mice was investigated (Figures 5A–C). For the remainder of the immunological studies, hexane extract was used owing to its superior effects. Anti-CD3/28 stimulated total lymphocytes had statistically significantly lower apoptosis than non-stimulated cells at all concentrations when treated with SSS. Still, SSS had a statistically significant (*p* < 0.05) lower apoptotic effect at 10^3^ concentrations when compared to CD3 alone (Figure 5B). Similarly, LPS-stimulated lymphocytes had significantly (*p* < 0.05) lower apoptosis when treated with SSS than unstimulated cells at all concentrations of SSS (Figure 5C). We also selected CD4^+^ T cells using CD4^+^ microbeads to observe SSS’s impact in the absence of antigen-presenting cells. In enriched CD4^+^ T cell cultures, activation-induced cell death (AICD) was higher than in total lymphocyte cultures, and SSS exposure did not impact AICD (Figures 5A–C). Collectively, these data argue for an anti-apoptotic role for SSS on murine lymphocytes and that this protection may be partially mediated by factors secreted by antigen-presenting cells.

### 3.5 Impact of SSS on cytokines produced from murine T cells in response to anti-CD3, PHA, and LPS mediated lymphocyte stimulation

**FIGURE 6:**
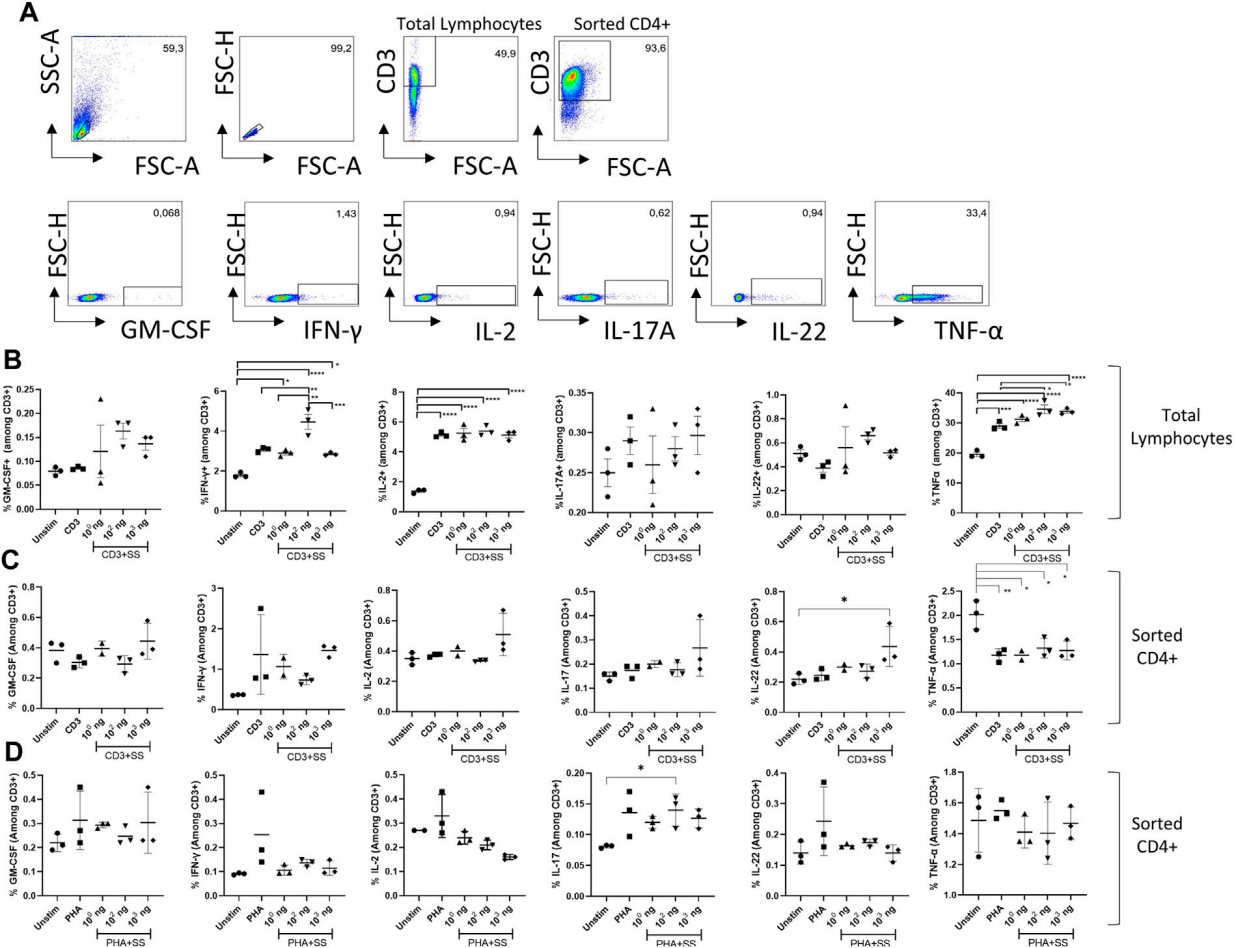
The impact of SSS on activation-induced cytokine production: **(A)** Flow cytometry gating strategy for T cells and cytokines quantified in the remainder of figure. **(B)** The percentage of GM-CSF, IFN-γ, IL-2, IL-17A, IL-22, and Tnf-α cytokines produced by T cells among lymph node-derived total lymphocytes upon activation with anti-CD3 (1 μg/mL). **(C)** The percentage of GM-CSF, IFN-γ, IL-2, IL-17A, IL-22, and Tnf-α cytokines produced by CD4+T cells microbead sorted from lymph node derived total lymphocytes and activated with anti-CD3 antibody (1 μg/mL) or **(D)** phytohemagglutinin (PHA) (10 μg/mL) for 24 h. The experiment was performed in technical triplicates with pooled lymphocytes from 1 mouse each time and repeated three times. (*) indicates *p* < 0.05, (**) indicates *p* < 0.01.

The effects of SSS extract on anti-CD3 or PHA stimulation-induced cytokine production in mouse lymph node-derived total lymphocyte cultures were examined (Figure 6). Gating startegy is given in (Figure 6A). Accordingly, although not statistically significant, a trend toward increase was observed in GM-CSF amounts at doses of 10^2^ and 10^3^ ng/mL compared to the unstimulated or SSS-free stimulation condition (Figure 6B). IFN-γ levels showed a statistically significant (*p* < 0.05) increase in frequency (expression), especially at 10^2^ SSS concentration.

In terms of IL-2 production, although a robust induction due to CD3/28 stimulation was observed, SSS exposure did not lead to a statistically significant change in IL-2 production by T cells (Figure 6B). Similarly, IL-17 and IL-22 production was not affected by varying doses of SSS exposure (Figure 6B). On the other hand, Tnf-α production was also induced by anti-CD3 stimulation, and SSS at concentrations of 10^2^ and 10^3^ ng caused a statistically significant increase in Tnf-α production compared to non-SSS stimulated cells (Figure 6B). These results indicate that SSS contributes to the production of more of this cytokine by affecting the transcriptional and post-transcriptional processes in the production of IFN-γ and Tnf-α cytokines by T cells after anti-CD3 activation. Because total lymphocytes contain antigen-presenting cells (dendritic cells\ macrophages, B cells monocytes) in addition to T cells, and the impact of SSS may be mediated through their effects on T cells, we also used microbead selected CD4^+^ T cells for the same experiments (Figures 6C, D). We stimulated the CD4^+^ T cells with either anti-CD3 (Figure 6C) or PHA (Figure 6D). Those experiments revealed upregulated IL-22 at high doses of SSS extract only in the anti-CD3 simulation condition and perhaps a non-significant upward trend in IFN-γ production. These results, combined with the apoptosis experiments with total lymphocytes and T cell enriched conditions, argue for the role of antigen-presenting cells in mediating the effects of SSS extract.

### 3.6 The effect of SSS on cytokine gene expression in murine lymphocytes

**FIGURE 7:**
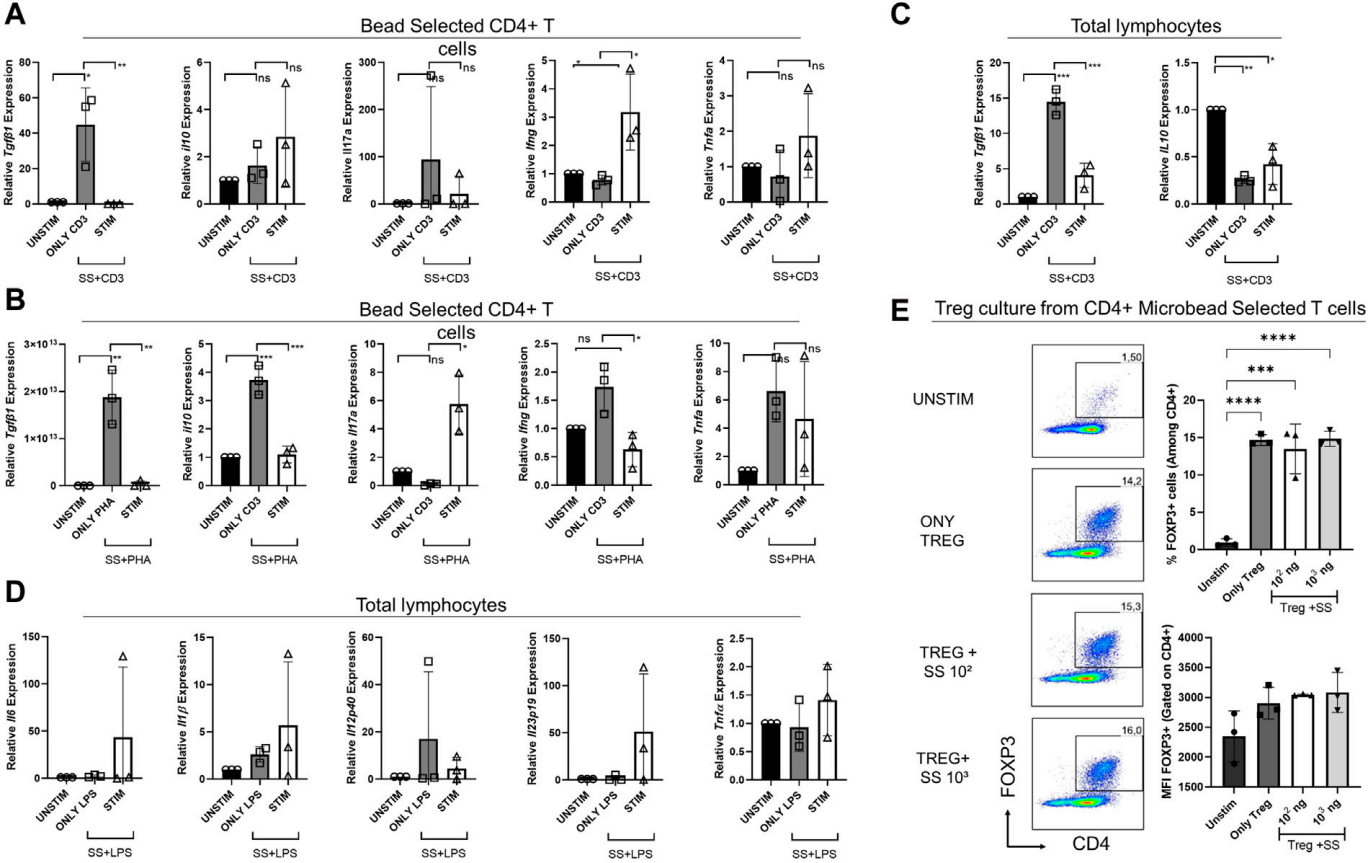
The impact of SSS on cytokine gene expression by sorted CD4^+^ T cells or total lymphocytes stimulated by anti-CD3 or PHA or LPS and Treg cell differentiation **(A)** Relative gene expression of cytokines *Tgfb, Il10*, *Il17a, Ifng, and Tnfa* produced by sorted CD4^+^ T cells stimulated with anti-CD3 or **(B)** PHA for 24 h and the gene expression was measured by Real-time qPCR. **(C)** Relative gene expression of cytokines *Tgfb* and *Il110*, produced by total lymphocytes stimulated with anti-CD3. **(D)** Relative gene expression of cytokines *Il6, Il1β, Il12p40*, *Il23p19*, and *Tnfα* produced by total lymphocytes stimulated with LPS for 24 h, and the gene expression was measured by Real-time qPCR. **(E)** Sorted mouse total CD4^+^ T cells were differentiated into Treg cells in the presence of recombinant Tfgβ, IL-2, and SSS extract at 100 and 1,000 ng/mL doses. Treg frequency (percent) and expression (Mean fluorescent intensity = MFI) were measured by Foxp3 staining. The experiment was performed in technical triplicates with pooled lymphocytes from a mouse. (*) indicates *p* < 0.05, (**) indicates *p* < 0.01.

We also examined the impact of SSS on the transcription of various cytokine genes via real-time qPCR (Figure 7). After anti-CD3 or PHA stimulation, either in total lymphocyte cultures or bead-selected CD4^+^ T cell cultures, *Tgfb* mRNA was consistently downregulated with the addition of SSS extract, marking TGFβ as the most consistently affected cytokine (Figures 7A–C). The Il17a, Ifng, and Tnfa mRNA data was inconsistent with anti-CD3 and PHA stimulation. However, bead-selected CD4^+^ T cells produced more *Ifng* when stimulated with anti-CD3, consistent with protein data. Similarly, an upward *Tnfa* message was observed, consistent with protein data. Lastly, in the PHA stimulation conditions, bead-selected CD4^+^ T cells produced significantly more *Il17a* mRNA (Figure 7B).

Due to differences in T cell responses in CD4^+^ T cell cultures, we investigated the impact of SSS extract on inflammatory cytokines produced in total lymphocyte cultures upon LPS stimulation, namely, Il6, Il1β, Tnfα, Il12p40, and Il23p19 (Figure 7D). The transcript levels of those cytokines did not differ significantly before and after SSS extract treatment.

The observation of lower Tgfb expression upon SSS extract exposure suggested that this very cytokine may have a toll on regulatory T cell (Treg) cell development or function, which is crucial in suppressing overt immune responses and immune homeostasis. Therefore, we sorted purified total CD4^+^ T cells from mouse lymph nodes and differentiated them into Treg cells *in vitro* (Figure 7E). Interestingly, SSS treatment did not negatively impact Treg induction, suggesting that *in vitro* recombinant Tgfb, present in in vitro cultures, may compensate for the lack of Tgfb and that *in vivo* experiments are needed.

These data suggest that SSS extract may have stimulus and cell-type-dependent immodulatory actions and that its most notable impact is the downregulation of Tgfβ.

## 4 Discussion and conclusion

Based on the studies conducted to date, it has been determined that snake skin contains keratin scales, cholesterol and its derivatives, α-keratin, fragile β-keratin, and many proteins and peptides. The presence of these compounds is thought to be remarkably effective in making snake skin highly elastic and giving it a unique structure (Vitt and Caldwell, 2013; Van Moorleghem et al., 2020; Andonov et al., 2023).

In a limited number of previous *in vivo* studies, SSS was found to affect acute wound healing through the release of inflammatory cytokines, epithelialization, neovascularization (Vakilian et al., 2019; Konop et al., 2021), and the reproductive cycle of mice through a hormone-cytokine exchange (Costa-Neto, 2005; Mukherjee et al., 2012). No *in vitro* studies have been performed with SSS in the literature. In this study, the SSS extracts obtained via different means were subjected to several different pharmacological and immunological tests to determine their possible anti-cancer and immune modulatory effects. This first *in vitro* study has yielded promising results on SSS’s antitumoral and pro-inflammatory activity.

The results of the MTT cytotoxicity tests revealed that the hexane extract of SSS was more effective in SK-MEL-30 cells than methanol and chloroform extracts and showed promising anti-cancer and anti-proliferative activity at low concentrations. Therefore, the primary conclusion of those assays is that the hexane extract of SSS may play a more effective antitumoral role at lower concentrations. As hexane SSS extract has shown the best effect, it was decided to use it, being non-apoptotic but having better anti-proliferative activity, for immunological flow cytometry studies.

The flow cytometry studies involving murine total lymphocytes supported the anti-apoptotic effect of SSS on immune cell lines. Apoptosis was significantly lower when SSS was added with anti-CD3/CD28. Similarly, LPS-stimulated cells had significantly (*p* < 0.05) lower apoptosis than unstimulated cells at all concentrations of LPS and SSS. These data suggest SSS exposure to lymphocytes allowed them to resist apoptosis in culture. In addition to T cells, antigen-presenting cells are present in the lymphocyte cultures. Therefore, the direct impact of SSS on CD4^+^ T cells was also measured. Unlike total lymphocyte cultures, SSS had no protective effect on CD4^+^ T cells in such cultures in response to activation-induced cell death. This suggests that non-T cells (possibly antigen-presenting cells) may be needed to mediate the protection observed in total lymphocyte cultures. With this study, the anti-apoptotic impact of SSS on lymphocytes has been demonstrated for the first time. Activation-induced T cell death is mediated by a mechanism involving Fas, Fas ligand (FasL)-mediated signaling pathway (Arakaki et al., 2014). Monitoring whether FasL and Fas expression decreases after CD3/CD28 activation or whether treatment with SSS causes a decrease in a proapoptotic Bid, Bad, Bax, or an increase in anti-apoptotic Bcl2 levels will help us understand the mechanisms by which T cells resist apoptosis. In addition to anti-CD3/CD28 stimulation, lymphocytes are more resistant to apoptosis under LPS stimulation. LPS stimulation mainly promotes cytokine production by antigen-presenting cells. In this condition, the formation of apoptosis resistance may indicate that the protective effect is also valid on cells other than T cells. Performing these experiments with different sorted cell populations and combining them with T cells separately will show whether resistance to apoptosis is generalizable to all cell types.

The effects of SSS extract on anti-CD3 or PHA stimulation-induced cytokine production in lymph node-derived lymphocytes and enriched CD4^+^ T cell cultures obtained from C57BL6 mice were examined. Accordingly, although not statistically significant, an upward trend was observed in GM-CSF amounts at doses of 10^2^ and 10^3^ ng/mL compared to the unstimulated or drug-free stimulation condition in total lymphocyte cultures.

SSS exposure did not affect IL-17 and IL-22 production by total lymphocytes or sorted CD4^+^ T cells. These interleukins are type 3 immune response-associated effector cytokines and play essential roles in developing protective immune responses against extracellular bacteria and fungi and lead to the mobilization of neutrophils and production of anti-microbial peptides and mucus (Rainard et al., 2020). It can be argued that SSS does not affect these cytokines and thus the type 3 immune response, but it should be kept in mind that these experiments were tested *in vitro* and specifically with exposure to the lymph node-derived cells. *In vivo* and especially mucosal tissue-specific investigations should be performed to support these results.

The most important result of anti-CD3/CD28 stimulation is the production of IL-2 cytokine responsible for T-cell proliferation (Verweij et al., 1991; Boulougouris et al., 1999). When the cells were evaluated in terms of IL-2 production, although an induction due to anti-CD3/28 stimulation was observed, SSS exposure did not lead to a statistically significant change in IL-2 production in either anti-CD3 or PHA-stimulation conditions regardless of pure CD4^+^ or total lymphocytes were used. Therefore, it can be concluded that SSS does not affect the TCR-mediated signaling pathway that triggers T cell activation and especially IL-2 production.

However, SSS exposure caused a statistically significant (*p* < 0.05) increase in IFN-γ frequency, especially at 10^2^ SSS concentration, and an insignificant increase by sorted CD4^+^ T cells activated by anti-CD3. One of macrophage activation’s essential cytokines, especially M1-type macrophages, is IFN-γ. Macrophage stimulation with IFN-γ induces direct antimicrobial and anti-tumor mechanisms and upregulates antigen processing and presentation pathways. In addition to IFN-γ enhancing natural killer (NK) cell activity (Mach et al., 1996; Carnaud et al., 1999) and regulating B cell functions such as immunoglobulin (Ig) production and classification, it regulates leukocyte movement. It directs many cell types’ growth, maturation, and differentiation (Finkelman et al., 1988; Boehm et al., 1997). These results support the hypothesis that SSS may have antitumoral features, primarily due to the effectiveness of this cytokine on NK cell function. IFN-γ is a pleiotropic molecule with associated anti-proliferative, proapoptotic, and anti-tumor mechanisms. It is generally recognized as the main factor of type I immunity. IFN-γ-mediated responses are positively related to patient survival in some cancers. However, although the success rate of IFN-γ-based therapies undergoing clinical trials is limited, IFN-γ signal insensitivity may play a protumorigenic role by leading to the downregulation of major histocompatibility complexes and upregulation of indoleamine 2,3-dioxygenase and checkpoint inhibitors (Castro et al., 2018).

Our data also showed that SSS at concentrations of 10^2^ and 10^3^ ng caused a statistically significant increase in Tnf-α production by total lymphocytes induced by anti-CD3 compared to non-SSS treated cells. Interestingly, Tnf-α production was affected by SSS only in total lymphocyte conditions, not by sorted CD4^+^ T cells. Given that a similar phenotype was observed in activation-induced cell death experiments, it suggests that either antigen-presenting cells are needed for this effect or CD8^+^ T cells may be affected by SSS preferentially. These results indicate that SSS may contribute to producing more of this cytokine by affecting the transcriptional or post-transcriptional processes in T cells after CD3 activation. The primary role of *TNF-*α, one of the cytokines involved in the acute phase reaction, is the regulation of immune cells. TNF can induce fever, apoptotic cell death, cachexia, and inflammation as an endogenous pyrogen. Still, it can suppress tumorigenesis and viral replication (Locksley et al., 2001) and promote dendritic cell activation and maturation to present tumor/and pathogen antigens. Thus, it is an essential mediator of immunomodulation in SSS.

We also tested the immune modulatory effects of SSS on endotoxin (LPS)-mediated (therefore antigen-presenting cell-mediated) cytokine induction by T cells (Supplementary Figure S1). Notably, TLR4 is mainly expressed by antigen-presenting cells (APC) (Kawasaki and Kawai, 2014). Its expression by T cells has been reported, yet its physiological role is unclear and appears different than in APCs (González-Navajas et al., 2010). Thus, we first aimed to explore the impact of SSS on indirect T cell activation by APCs by probing T cell-derived cytokines. SSS had no significant effect on GM-CSF production, whereas IFN-γ production was induced only at a dose of 10^2^, as in anti-CD3/CD28 stimulation, leading to a high IFN-γ production. On the other hand, in contrast to anti-CD3/CD28 stimulation, IL-2 production was downregulated in the presence of SSS when LPS stimulation was performed, although not reaching statistical significance. Under LPS stimulation conditions, IL-17A, IL-22, and Tnf-α were upregulated, although not reaching statistical significance, especially at the dose of 10^3^ SSS compared to the non-SSS group. These protein levels data suggest that SSS may have a pro-inflammatory role, most significantly marked by the production of Tnf-α and IFN-γ.

To assess the effect of SSS at the transcription level, gene expression levels *of Ifng, Il17A, Il10,* and *Tgfβ* after anti-CD3 or PHA stimulation and *Il6, Il1β, Tnfα, Il12p40, Il23p19* cytokines under LPS stimulation conditions were also investigated. Under anti-CD3 stimulation conditions, a significant increase in *Ifng* transcript level was observed in the SSS-treated sorted CD4^+^ T cells (Salerno et al., 2019). Interestingly, SSS caused a dramatic decrease in *Tgfb* expression in anti-CD3 or PHA stimulation conditions by CD4^+^ T cells and total lymphocytes. The reduction in the suppressive cytokines appeared to be *Tgfb*-specific, as SSS altered the levels of *Il10* transcript only in CD4^+^ T cells with anti-CD3 stimulation but not PHA nor in the total lymphocyte cultures.

Our data also revealed that SSS did not significantly impact induced Treg cell generation/expansion *ex vivo*. This finding is important because Tgfβ is required for Treg cell development and suppressive functions (Dikiy and Rudensky, 2023). However, more *in vivo* studies are needed to determine the impact of SSS extract on Treg cells unequivocally. *In vitro*, Treg differentiation cultures are supplemented with recombinant Tgfβ. Therefore, the recombinant cytokine added to the culture could compensate for the lack of this cytokine. *In vivo* studies or *in vitro* suppression assays will be more informative for future studies.

Although our data shows no effect on the LPS-stimulated conditions, no statistically significant results were observed for the cytokines analyzed. The reduced *Tgfb* gene expression results and the increases in Ifng and Tnfα protein levels collectively support a pro-inflammatory role of SSS.

Studies on SSS’s potential medical and pharmacological utility *in vivo* are minimal. In this first *in vitro* study, we provide experimental evidence to support a partial antitumorigenic and pro-inflammatory activity for SSS on cell lines and murine lymphocytes. The data presented herein warrant further investigations in future studies, which will shed light on SSS’s *in vivo* antitumoral or pro-inflammatory activity. In addition, detailed studies are needed to identify the molecule or molecules mediating these anti-tumor and pro-inflammatory effects in SSS extract.

## Data Availability

The original contributions presented in the study are included in the article/Supplementary Material, further inquiries can be directed to the corresponding author.
