# Supplementary figures and images for: Investigation of immunomodulatory and cytotoxic effects of shed snake skin (Elaphe sauromates) extract
Source: Front Pharmacol. 2024 Jul 12;15:1270970. doi: 10.3389/fphar.2024.1270970 (PMC11272602; doi:10.3389/fphar.2024.1270970)

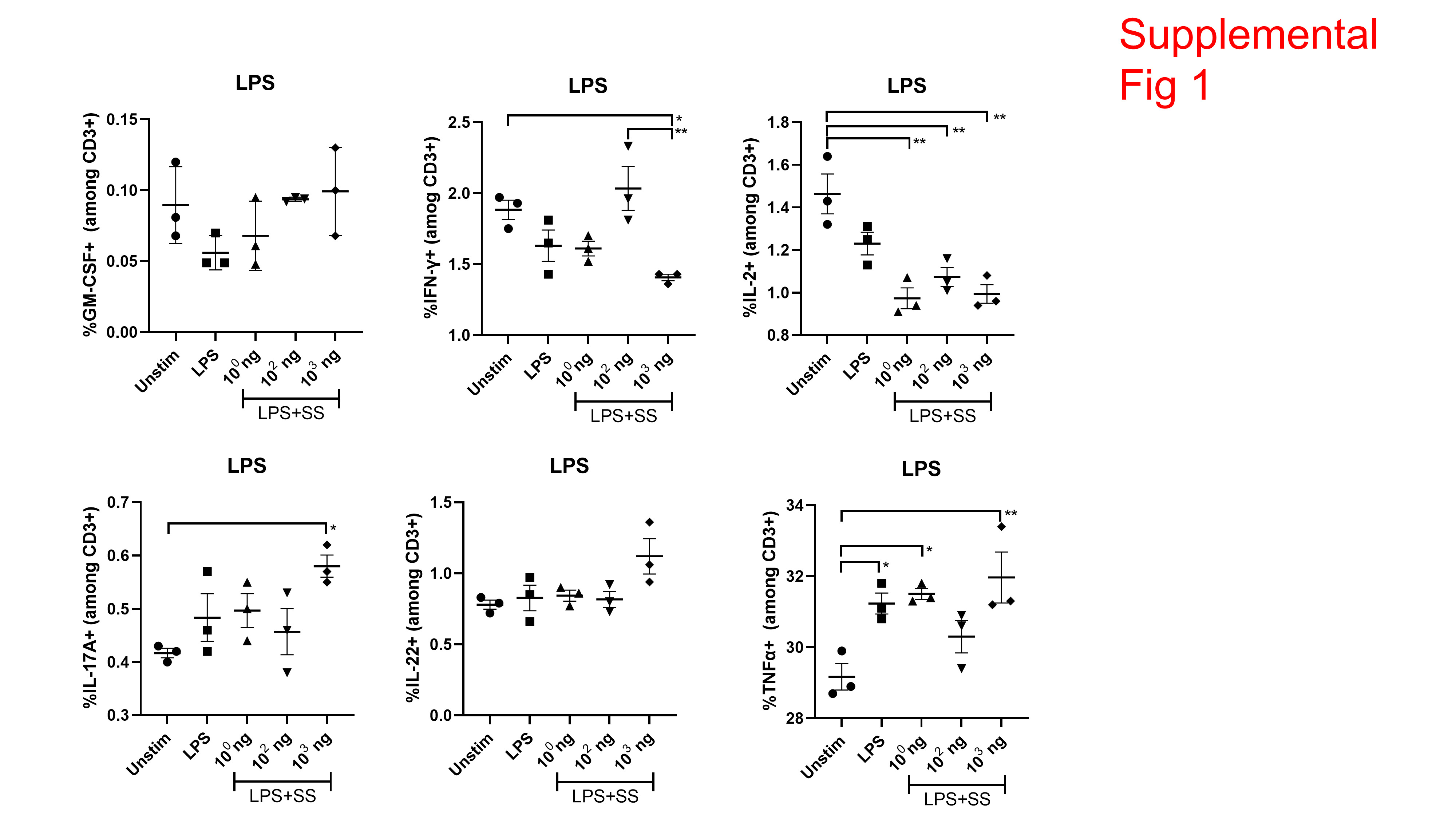

Supplement: Supplementary file 1 [file Image1.JPEG]
